# The relationship between staffing and adverse events in Washington State hospitals: a cross-sectional study using linked hospital data

**DOI:** 10.21203/rs.3.rs-3979968/v1

**Published:** 2024-02-27

**Authors:** Nathaniel D. Blair-Stahn, Kyla F. Woodward, Sarah J. Iribarren, Alix Pletcher, Natalie Hoge, Abraham Flaxman

**Affiliations:** UW Institute for Health Metrics and Evaluation; UW School of Nursing; UW School of Nursing; UW Institute for Health Metrics and Evaluation; UW School of Nursing; UW Institute for Health Metrics and Evaluation

**Keywords:** staffing, patient outcomes, workforce, care team

## Abstract

**Objective:**

To quantify the relationship between staffing characteristics and patient outcomes in acute care hospitals in Washington state.

**Methods:**

Retrospective cross-sectional time-series study of linked data from six sources on staffing and outcomes for Washington state hospitals. Key stakeholders provided input on data sources, measures, and outcomes in a four-phase participatory process. After data cleaning and linkage, we used a random effects Poisson regression model to examine the relationship between staffing levels or characteristics and adverse outcomes.

**Results:**

The study included 263 hospital-years from 80 distinct hospitals, with 162 hospital-years from general acute care hospitals (n=46) and 101 hospital-years from critical access hospitals (n=34). In general acute care hospitals, a higher ratio of patients to care team staff is associated with a higher number of adverse events (adjusted RR, 1.36 per one SD increase; 95% UI 1.13–1.63), and a lower proportion of RNs on the care team staff is likely associated with a higher number of adverse events (adjusted RR, 1.16 per one SD increase; 95% UI, 0.97–1.39). In critical access hospitals, a lower proportion of RNs on the care team is associated with a higher number of adverse events (adjusted RR, 3.28 per one SD increase; 95% UI, 1.20–7.75). A counterfactual analysis indicated that if all general acute care hospitals had no more than the median staffing ratio of 1.2 patient hours per staff hour, the number of adverse events would be reduced by 10% (95% UI 2.7–16.8).

**Conclusion:**

RN staffing is an indisputable component of safe, high quality patient care, and other factors such as availability of care team staff, hospital features, and patient characteristics also impact patient outcomes. This study highlights the utility of merging diverse data sources to provide a comprehensive analysis of the relationships between staffing and patient outcomes.

## INTRODUCTION

The relationship between registered nurse (RN) staffing and patient outcomes in the acute care setting has been well established, with multiple studies showing that increasing the number of patients an RN cares for results in poorer patient outcomes.[1–6] RNs provide care in complex settings where community and organizational features influence the resources, personnel, and equipment available. Low staffing may reflect organizational issues such as work environment, pay and promotion practices, and other structures that diminish recruitment and retention of RNs.[7] RN education and experience, patient acuity, availability of supplies and medications, and staffing levels of other care team members (e.g. respiratory therapy or environmental services) may also mediate the relationship between RN staffing and patient outcomes in hospitals. [8] Data suggest that in the absence of resources and personnel, RNs take on additional non-nursing duties, taking time away from nursing work[8] and impacting patient outcomes.[2, 9] Other issues impacting care include community features like access to preventive healthcare, tertiary referral center proximity, housing for healthcare workers, and population features such as social determinants of health that affect acute and chronic health conditions; geography and rurality also influence staff availability, patient needs, and hospital logistics.[8] For example, some rural hospitals are designated as Critical Access Hospitals (CAHs) by the Center for Medicare and Medicaid Services and characterized by 25 or fewer inpatient beds, location more than 35 miles from another hospital, and continuous availability of emergency services.[10] Despite these contextual factors, few studies look beyond RN staffing to evaluate the impact of other elements.[11]

**Figure 1:**
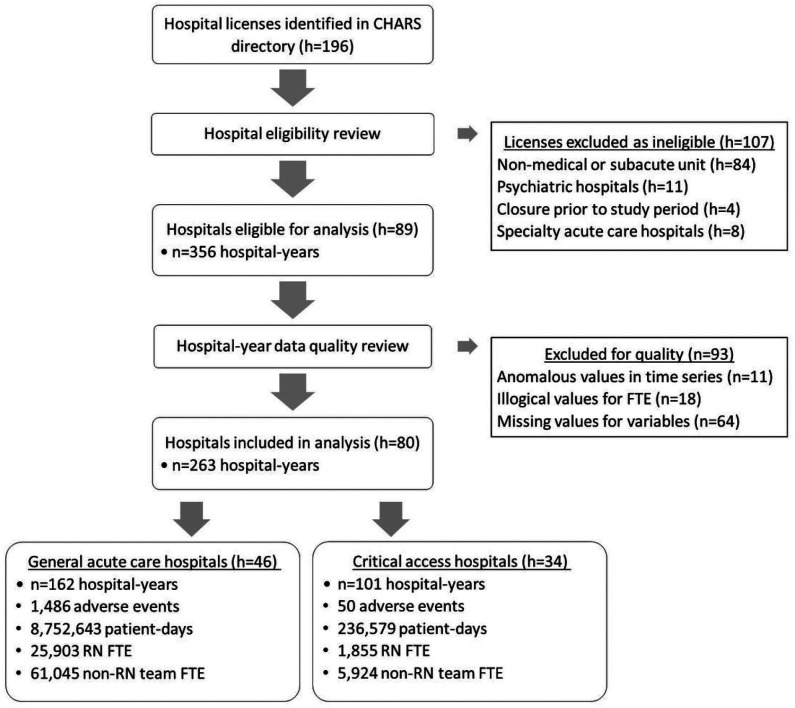
Data selection flowchart.

In recent years, the Washington state (WA) legislature considered legislation promoting safe levels of RN staffing in hospitals. Because there were no current data examining staffing and patient outcomes within the state, the 2021 legislature directed the WA Department of Health to contract a team of University of Washington researchers to conduct a study examining the impacts of healthcare staffing composition and characteristics on patient outcomes.[12] Our team developed the study in 4 phases. In the first phase, we performed a review of reviews to examine commonly used variables and how they were defined or operationalized in previous research.[11] Next, we developed a causal model (Fig. 1) linking staffing to patient outcomes based on phase 1 findings (phase 2), followed by a participatory process of model refinement using feedback from stakeholders across the state[8] as well as exploring and obtaining stakeholder input on available statewide data sources that allowed us to examine specific variables of interest (phase 3). The present study reports the fourth and final phase, where we used the refined causal model and merged datasets to analyze the impact of staffing on adverse patient outcomes, accounting for hospital and patient characteristics. In this process, we developed and tested four hypotheses: H1) an increased ratio of patients to care team staff is associated with a higher number of adverse events; H2) a lower proportion of RNs among care team staff is associated with a higher number of adverse events; H3) a lower proportion of baccalaureate-prepared RNs is associated with a higher number of adverse events; and H4) a higher proportion of RNs with less than 5 years of experience is associated with a higher number of adverse events.

## METHODS

In this retrospective cross-sectional time series study, we linked data from six state-level sources to compare staffing characteristics and patient outcomes for hospitals across Washington. Throughout the study, we used feedback from key stakeholders to refine the selection of participants, data sources, analysis, and outcomes.

### Sample

We identified hospitals using license numbers and data in the 2021 Comprehensive Hospital Abstract Reporting System (CHARS) directory from the WA Department of Health. Eligible hospitals were Washington acute care hospitals (including CAHs) in operation for at least part of the study period (2016–2019). Exclusion criteria were identification as a specialty hospital (psychiatric, rehabilitation, cancer care, or pediatric) or wing such as an extended care unit. We chose the study period based on stakeholder feedback that the years before the COVID pandemic needed to be examined separately from the years after the onset of COVID. We conducted a complete case analysis by excluding all data points for which any of the model variables were missing.

### Data sources and processing

During interviews and a townhall meeting, we asked key stakeholders to provide input on existing state level data sources and the strengths and limitations of those sources, then we screened available sources and selected based on data compatibility and applicability. Sources included the CHARS, individual hospitals’ year-end reports to the Department of Health, adverse event data from the Department of Health, nursing licensure information from the Board of Nursing (formerly the Nursing Care Quality Assurance Commission), and other publicly available records. See Supplement 1 for a complete listing of sources and variables. The standard unit of measurement was a hospital-year, which consists of the merged data containing all model variables pertaining to a single hospital for a single year. Each data source required processing to link it to other data. For example, year-end reports contained cost centers specifying nursing and non-nursing full time equivalents (FTEs). We aggregated these data to indicate the total number of nursing or other care team FTEs in a facility for each hospital-year. Nursing licensure data includes reports of RNs’ employers’ zip codes, so we matched these with the unique organization corresponding to that zip code, then aggregated data to determine model variables for education and experience in a given hospital. CHARS data include complex patient-level information based on discharges and required the most processing to assign outcomes and aggregate and measure covariates per patient-days and hospital-years.

### Outcomes

The primary outcome of interest was the number of serious adverse events, which we defined to include reportable pressure ulcers, falls, and medication errors. While stakeholders identified multiple pertinent patient outcomes in the model (Fig. 1), adverse events are commonly used as care quality indicators related to staffing and nursing care.[13] We identified the number of events for each hospital year by aggregating data from state-level reports which follow the National Quality Forum 2011 definitions of reportable events.[14] Adverse events represented in this study include stage 3, stage 4, or unstageable pressure ulcers acquired after admission to the healthcare setting and patient death or serious injury associated with either a medication error or a fall during inpatient admission. The outcome variable we used in our model was the total number of these three specific types of adverse events reported by each hospital during each year of the study.

### Exposure variables

Our four exposure variables corresponded to four of the staffing characteristics requested by the legislature: number, type, education, and experience. Our variable for staff number was the patient-to-staff ratio, defined as the total number of inpatient hours recorded in CHARS divided by the number of care team staff hours converted from FTE as recorded in the year-end cost center reports. Care team staff consisted of acute care nursing staff plus core clinical and nonclinical staff. Nurses on the research team (SI) and among the key stakeholders collaborated to determine these categories based on examination of available cost centers (Supplement 2). Our other three exposure variables were the ratio of RN FTEs to all care team staff FTEs (staff type), the fraction of RNs with less than 5 years since licensure (experience), and the fraction of RNs without a Bachelor of Science in Nursing (BSN) (education).

### Control variables

Our desk review, key informant interviews, and town hall feedback identified a complex causal web of factors that might confound the relationship between staffing and outcomes; the following briefly describes the covariates we included to control for this within the model categories (Fig. 1.).[8, 11] Hospital characteristics included trauma level designation, percent of patients transferred in from other hospitals, and certification for open heart surgery, which can be used as a proxy for equipment and technology.[5] Patient characteristics included demographics such as age, sex (recorded as female, male, or unknown by hospital standards[16]), race (recorded per Office of Management and Budget standards[15]), and Medicaid insurance status. Other patient characteristics included the hospital’s case mix index for the year, the average number of diagnoses in a hospital-year, the percentage of patient-days with an obstetrics procedure (ICD-10-PCS code starting with 1), and the percentage of patient-days with a high care intensity diagnosis or procedure code. Based on stakeholder feedback, we defined high care intensity diagnoses as dementia, self-harm, opioid use, drug dependency, obesity, traumatic brain injury, heart failure, and asthma using ICD-10-CM diagnosis codes in CHARS. Stakeholders reported that diagnoses alone were insufficient indicators of care intensity, so we worked with several nurses to review the 500 most common ICD-10-PCS procedure codes in CHARS and rated them as likely leading to higher intensity care versus usual care intensity (Supplement 3).

Finally, we conducted separate analyses for CAHs and general acute care hospitals (ACHs), which we defined to be any eligible acute care hospital that was not designated as a CAH in the CHARS directory. This decision was based on stakeholder feedback that the vastly different staffing models and requirements for CAHs[10] meant that staffing did not vary as widely in those settings as in ACHs.

### Statistical analyses

For each category of hospital (ACH, CAH), we fit a hierarchical Bayesian Poisson regression model predicting the number of adverse events in each hospital-year as a function of our exposure and control variables. We obtained little data for hospital-level control variables, so we included hospital-level random effects to account for similarities between measurements at the same hospital in different years. The model has the following form:

$$\text{adverse}\_\text{event}\_{\text{count }}_{h,y}\sim \text{Poisson }\left(\text{exp}\left(\mu_{h,y}\right)\times {\text{patient\_days }}_{h,y}\right)\text{, }$$

$$\mu_{h,y}=\alpha +X_{h,y}\cdot \beta +u_{h},$$

with priors

$$\alpha \sim \text{Normal}(0,500),\;u_{h}\sim \text{Normal}\left(0,\sigma^{2}\right),$$

$$\beta_{i}\sim \text{Normal}(0,10),\;\sigma \sim \text{HalfNormal}(100).$$

with priors

$$\alpha \sim \text{Normal}(0,500),\;u_{h}\sim \text{Normal}\left(0,\sigma^{2}\right),$$

$$\beta_{i}\sim \text{Normal}(0,10),\;\sigma \sim \text{HalfNormal}(100).$$

Here, *h* indexes hospitals, *y* indexes years, and *i* indexes the explanatory variables in the model (exposures plus controls). *X* is the hdesign matrix, with each column (indexed by *i*) corresponding to an explanatory variable, and each row (indexed by (*h,y*)) corresponding to one hospital-year of data. Hospital-level random effects are represented by *u*_*h*._ The model estimates values for σ *u*_*h*_ α, and the vector *β* = (*β*_1_, …, *β*_K_) where *K* is the total number of explanatory variables.

The model assumes a log-linear relationship between the explanatory variables and the expected rate of adverse events per patient-day. In particular, exp (*β*_*i*_)is the proportional increase in the expected adverse event rate when exposure variable *i* increases by one unit; we call exp (*β*_*i*_) the *adjusted rate ratio* per unit increase in exposure variable *i*. Because of the structure of our causal diagram, this interpretation as an adjusted rate ratio applies to the *exposure* variables (staffing characteristics), whose effects have been adjusted for confounding as much as possible given our data, but not to the control variables (hospital and patient characteristics), which may still have confounding present.

To estimate the *unadjusted* rate ratio for each exposure variable, we fit a univariate Poisson regression of the same form but without random effects. This form of the model estimates α and a single *β*_*i*_, which can be exponentiated to obtain the unadjusted rate ratio for the corresponding exposure variable.

All regressions were done using NumPyro version 0.10.0 with Python 3.9.12. All explanatory variables were standardized to have mean 0.0 and standard deviation (SD) 1.0 before fitting the regression models. Thus, each exp (*β*_*i*_)value from each model is an estimate of the increase in adverse event rate ratios per one SD increase of the corresponding exposure variable.

### Sensitivity and counterfactual analyses

To confirm that the findings were robust to the sample of hospitals, we conducted out-of-sample validation. We re-ran our analyses 10 times with 10% of the hospitals randomly removed from the data. This resulted in no substantive change in our findings. We also completed a counterfactual analysis to present findings to stakeholders in more meaningful terms. We used our fitted random effects Poisson regression to predict how many adverse events would be averted in a hypothetical scenario in which all ACHs had the median patient-to-staff ratio or lower (with all other variables remaining the same). We chose the median of the observed data as a level that could be realistically achieved and that fell within the range of our models’ predictions.

## RESULTS

### Hospitals included in study

**Figure 2:**
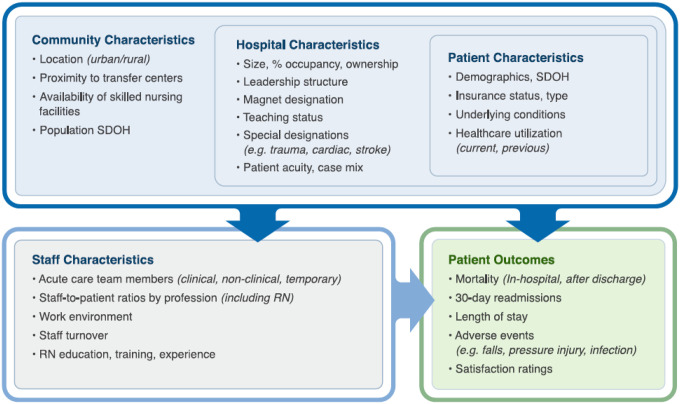
The WACCHO model of community, hospital, patient, and staffing characteristics that impact patient outcomes.

The CHARS 2021 hospital directory contained 196 license numbers. Of these, 101 were acute care licenses, of which 4 had closed and 8 were specialty facilities (see Fig. 2). This left 89 eligible hospitals and 356 hospital-years. During data cleaning, we excluded 93 hospital-years due to anomalous, illogical, or missing values. In total, our analysis included 263 hospital-years for 80 distinct hospitals: 162 hospital-years from 46 AHCs and 101 hospital-years from 34 CAHs.

During the study period, 1,486 adverse events occurred at ACHs, and 50 occurred at CAHs. The higher number of events in ACHs is explained both by the larger sample size and the fact that CAHs are limited to 25 beds. Dividing by the sample sizes, the mean yearly rate of adverse events was 9.17 in ACHs and 0.50 in CAHs. Table 1 shows descriptive statistics for each model variable across all hospital-years for the two hospital types. Focusing on the four exposure variables, the mean patient-to-staff ratio was larger at ACHs than at CAHs (1.20 vs. 0.37), as was the fraction of BSNs, while the fractions of RN FTEs and of new RNs were similar between the two hospital types.

### Association between staffing characteristics and adverse events

Due to the differences between ACHs and CAHs, we performed separate analyses of our four hypotheses for each hospital type. All reported adverse event rate ratios (RRs) are per one standard deviation (SD) increase of the specified exposure variable.

For ACHs, we found that a higher ratio of patients to care team staff is associated with a higher number of adverse events (H1). The adjusted RR was 1.36 (95% UI, 1.13–1.63), meaning that if the patient-to-staff ratio were to increase by one SD relative to all ACHs in WA, we would expect the number of adverse events per patient-day to increase by a factor of 1.36 on average. We also found that a lower percentage of RNs on the care team staff is likely associated with more adverse events (adjusted RR, 1.16; 95% UI, 0.97–1.39), but the uncertainty interval includes the possibility of no effect (H2). Our findings did not strongly support the hypotheses that a lower proportion of BSNs or a higher proportion of RNs with less than 5 years of experience were associated with higher numbers of adverse events in ACHs (Table 2) (H3, H4).

For CAHs, we found that a lower proportion of RNs on the care team is associated with a higher number of adverse events (adjusted RR, 3.28; 95% UI, 1.20–7.75) (H2). Our results for CAHs were much more uncertain than the results for ACHs, and they did not support our other three hypotheses (H1, H3, H4) (Table 2). Instead, the adjusted RR for the fraction of RNs with less than 5 years of experience was 0.45 (95% UI, 0.11–1.07), suggesting that morefinew RNs on the care team may be associated with *fewer* adverse events in CAHs (H4). However, the uncertainty interval includes the possibility of no effect, and the fraction of new RNs could be acting as a proxy for some other, unmeasured variable.

### Counterfactual analysis

The counterfactual analysis indicated that if all ACHs averaged no more than 1.2 patient hours per staff hour (the median patient-to-staff ratio in WA during the study period), then the number of adverse events would be reduced by 10% (95% UI 2.7–16.8). This means that among the 1,486 adverse events that occurred between 2016–2019, approximately 149 could have been averted by ensuring that all ACHs had sufficient staffing to guarantee at most 1.2 patient hours per hour worked by acute care team staff.

## DISCUSSION

Our findings confirm two of our hypotheses about the relationship between staffing and patient outcomes. The finding focused on RN staffing confirms what is known from prior research,[1–6] while the other related to acute care team staffing more broadly suggests a gap in current knowledge that requires more thorough exploration. The two hypotheses that were not confirmed relate to RN education and experience, and the process of testing those hypotheses revealed the difficulties in assessing staffing characteristics among multiple organizations at a granular level. This discussion will focus on the gap in knowledge suggested by findings and the limitations of current data to answer some workforce and staffing questions.

### Acute care staffing

Among ACHs, the primary finding was that a higher patient-to-care-team-staff ratio is associated with increased adverse events. This finding aligns with previous research on the relationship between RN staffing and patient outcomes, but the absence of a significant finding related to the proportion of RNs in this group suggests that RN staffing is not the only driver of patient outcomes. Many departments in the hospital support patient care. Worker shortages in these areas can lead to operational failures; for example, a shortage of central supply workers means that patient supplies are missing from the unit, affecting staff and patient satisfaction and the quality of care.[17] In these instances, nursing staff may step in to complete extra tasks, but the more tasks an RN takes on, the less time they have available to complete nursing work more aligned with their scope of practice, resulting in missed nursing care and increased burnout.[2] In a nationwide workplace-focused survey in 2022, among 5,461 acute care RN respondents to a question about the availability of appropriate ancillary staff, 27% rated availability as ‘seldom’ or ‘never’, 39% as ‘sometimes’, and only 29% rated availability as ‘often’ or ‘always’.[18] These results suggest that RN staffing ratios need to be examined in the broader context of appropriate staffing across multiple departments that support patient care both directly and indirectly, a theme also highlighted in our qualitative study.[8] Additional research needs to rigorously address staffing in other core acute care areas and evaluate its impact on patient outcomes.

### RN experience and education

We found no association between RN education or experience and adverse events. However, the analysis of these measures was limited by the variability of available data and overall lack of granularity. For example, an RN with 10 years of experience may have only 1 year working in an acute care setting or on a specific type of unit, but this level of detail is not available in the licensure data. RN education data often reports the nursing degree associated with initial licensure but does not include additional classes, certifications, or training which may provide the RN with specialty skills. In addition, these types of training are not consistently gathered or reported at the organizational or state level, making it difficult to link them with patient outcomes. Previous studies examining the impact of RN education level frequently use patient mortality rather than adverse events as the primary outcome, so exploring associations between education and adverse events may bear different results. [19] Rather than suggesting a lack of relationship between RN characteristics and patient outcomes, our findings highlight the difficulty in obtaining data that can match staff characteristics with patient outcomes at a granular level. One mechanism to improve tracking of individual RNs is use of a National Provider Identifier, which provides a unique Identifier allowing linkage of education, specific types of training or certification, and patient care activities.[20]

### Limitations

In general, our findings are limited by the challenges in measuring and defining variables among multiple hospitals with varying data collection practices. Individual organizational features such as process improvement work were not captured in the data, nor were more granular features such as equipment or technology available to indicate an organization’s ability to handle higher acuity patients or more complex procedures. One main limitation was the way that staffing FTE are reported. Hospitals provided numbers of FTEs in different cost centers without differentiation of staff types in those cost centers. For example, certified nurse assistants are not reported separately within cost centers, nor are the numbers of contract staff working on specific units, and different organizations may have placed staff in categories differently. Variables such as experience and training are available for RNs due to state licensing surveys, but not for other staff members. Patient outcomes relied on reported adverse events, which may underrepresent overall events and does not include other measures of patient care quality such as guideline adherence, and there is no established diagnostic or treatment codes that may indicate a patient requires complex, technical care. Despite these limitations, we pulled together diverse data sources to create a merged dataset that allowed us to complete a robust analysis and identify known relationships between hospital characteristics, staffing, and outcomes in acute care settings.

## CONCLUSION

We need a robust understanding of the complexities of the acute care environment to develop and implement effective staffing policies. RN staffing is an indisputable component of safe, high quality patient care, but it is joined and mediated by other factors such as availability of other care team staff, resources, and hospital and patient characteristics. This study highlighted the utility of merging diverse data sources to provide a more comprehensive analysis of the relationships between staffing and patient outcomes. Improving statewide data collection and staffing reporting practices would provide additional opportunities for more granular examination of links between staffing and patient outcomes. Developing policies that focus on care team staffing in addition to RN staffing will offer opportunities to improve patient care more effectively while protecting RNs from the high demands and burnout associated with doing non-nursing work.

## Figures and Tables

**Table 1:** Descriptive statistics of hospital-years included in analysis. Shown are the mean, standard deviation (SD), and range of each variable in the model. For the two binary variables, we show the count and percentage of hospital-years with value 1 instead of the mean and SD.

	General acute care hospitals (n = 162 hospital-years)		Critical access hospitals (n = 101 hospital-years)	
Model variable	Mean (SD)	Min–Max	Mean (SD)	Min–Max
Outcome variables				
Number of adverse events	9.17 (10.39)	0–49	0.50 (0.89)	0–5
Staffing characteristics				
Patient-to-staff ratio	1.20 (0.40)	0.02–2.31	0.37 (0.30)	0.00–2.57
% Non-RN FTE on care team	71.7 (9.1)	46.2–96.7	72.8 (14.9)	28.1–98.0
% RNs without BSN	41 (17)	0–100	61 (20)	25–100
% RNs with < 5 years since licensure	28 (15)	0–64	21 (19)	0–100
Hospital characteristics				
% Transfer patients	7.3 (8.8)	0.0–39.3	1.4 (5.8)	0.0–48.0
Count of hospital-years with trauma level I or II	26 (16.0%)	–	0 (0.0%)	–
Count of hospital-years with capacity for open heart surgery	51 (31.5%)	–	0 (0.0%)	–
Patient characteristics				
Case mix index	0.99 (0.27)	0.45–2.13	0.69 (0.16)	0.46–1.30
Average number of diagnoses	11.2 (2.3)	4.1–17.3	9.9 (3.2)	3.3–16.9
% High-intensity patient-days	63.6 (9.6)	26.3–79.0	42.9 (11.9)	13.1–65.5
% Obstetrics patient-days	4.0 (3.0)	0.0–16.6	5.0 (5.9)	0.0–19.8
Average patient age	51.2 (7.2)	26.5–68.8	56.5 (12.5)	32.7–82.5
% Male patients	46.5 (4.8)	33.7–64.8	43.1 (6.8)	22.8–75.0
% Non-white patients	12.2 (8.4)	0.0–33.5	5.6 (9.7)	0.0–72.4
% Medicaid patients	35.1 (13.1)	1.2–83.7	32.3 (15.1)	0.0–84.2

**Table 2:** Effects of staffing characteristics on adverse events. Unadjusted and adjusted rate ratios (RRs), with 95% uncertainty intervals (UIs), show the proportional increase in the mean adverse event rate when the exposure variable increases by one standard deviation (SD).

Exposure variable	Unadjusted RR per SD increase (95% UI)	Adjusted RR per SD increase (95% UI)
General acute care hospitals		
Patient-to-staff ratio	1.04 (0.97–1.10)	1.36 (1.13–1.63)
% Non-RN FTE on care team	1.16 (1.09–1.22)	1.16 (0.97–1.39)
% RNs without BSN	0.95 (0.90–1.00)	1.18 (0.91–1.52)
% RNs with < 5 years since licensure	0.99 (0.95–1.04)	1.08 (0.88–1.33)
Critical access hospitals		
Patient-to-staff ratio	0.85 (0.60–1.08)	0.98 (0.49–1.88)
% Non-RN FTE on care team	1.88 (1.25–2.80)	3.28 (1.20–7.75)
% RNs without BSN	1.13 (0.80–1.55)	1.88 (0.59–4.81)
% RNs with < 5 years since licensure	0.92 (0.57–1.36)	0.45 (0.11–1.07)

## What is known

- The RN-to-patient staffing ratio and RNs’ educational preparation impact patient care outcomes such as morbidity and mortality in the acute care setting

### What this study adds:

- Staffing of acute care team members overall and the proportion of RNs among staff impact the number of reportable adverse events in acute care settings.

### How study might affect research/practice/policy:

- Further research in the impact of staffing on care outcomes should include other professions beyond nursing.
- Policy initiatives to improve staffing should take into account care team roles in addition to RNs and specific features of acute care hospitals that may change staffing needs.
- Community, organizational, staff, and patient factors all need to be considered when examining the relationship between staffing and patient outcomes.
